# Active surveillance as a management strategy for papillary thyroid microcarcinoma

**DOI:** 10.20892/j.issn.2095-3941.2019.0470

**Published:** 2020-08-15

**Authors:** Huan Zhang, Xiangqian Zheng, Juntian Liu, Ming Gao, Biyun Qian

**Affiliations:** ^1^Cancer Prevention Center, Tianjin Medical University Cancer Institute and Hospital, National Clinical Research Center for Cancer, Key Laboratory of Cancer Prevention and Therapy, Tianjin, Tianjin’s Clinical Research Center for Cancer, Tianjin 300060, China; ^2^Department of Head and Neck Tumor, Tianjin Medical University Cancer Institute and Hospital, National Clinical Research Center for Cancer, Key Laboratory of Cancer Prevention and Therapy, Tianjin, Tianjin’s Clinical Research Center for Cancer, Tianjin 300060, China; ^3^Hongqiao International Institute of Medicine, Shanghai Tongren Hospital and Faculty of Clinical Research Center, Shanghai Jiao Tong University School of Medicine, Shanghai 200025, China

**Keywords:** Papillary thyroid carcinoma, microcarcinoma, active surveillance, guidelines, low risk

## Abstract

Active surveillance (AS) can be considered as a treatment strategy for low risk papillary thyroid microcarcinoma (PTMC), with the absence of clinically apparent lymph nodes, extrathyroidal extensions, and distant metastasis. After reviewing the reports on AS of low risk PTMCs worldwide, we introduced AS, and discussed the selection criteria for active surveillance candidates based on different guidelines and the follow-up schedules. Moreover, the requirement of cytological diagnosis, progression evaluation methods, necessity of thyrotropin suppression, and medical costs were issues that both clinicians and patients considered. The usefulness of AS for low risk PTMC patients depended on accurate and confidential evaluation of patient risk. Clinicians may adopt measures like dynamic monitoring, risk stratification, and making personal follow-up schedules to minimize these potential risks. By appropriately selecting PTMC patients, AS can be an effective alternative treatment to immediate surgery.

## Introduction

Papillary thyroid microcarcinoma (PTMC) is defined as papillary thyroid carcinoma with the largest diameter of ≤ 10 mm. Some PTMCs have aggressive features, such as clinical node metastasis, distant metastasis, and invasive symptoms to the recurrent laryngeal nerve or trachea, while other PTMCs without these aggressive features are low risk and slow growing. The aggressive management of PTMC usually involves immediate surgery. However, low risk nonaggressive PTMC has other options besides immediate surgery. In this review, we focused on active surveillance (AS), which is a management strategy of PTMC involving a low risk.

AS as a strategy for low risk PTMC was first initiated in Japan. AS was incorporated into guidelines of Japan in 2010 and the USA in 2015. The Japan Association of Endocrine Surgeons (JAES) and the Japanese Society of Thyroid Surgeons (JSTS) established the first edition of guidelines of differentiated thyroid carcinomas in 2010, which adopted AS as an option for low risk PTMC^1^. The 2015 guidelines of the American Thyroid Association (ATA) also incorporated AS as a management strategy for low risk PTMC^2^.

## Epidemiological characteristics of PTMC

Despite the differences in incidences and mortalities of thyroid cancers between countries, the worldwide incidence of thyroid cancer has increased over the past 50 years. In the USA, thyroid cancer is one of the fastest growing cancers, whose incidences increased from 4.9 per 100,000 in 1975 to 14.3 per 100,000 in 2009^3^. The data from the Surveillance, Epidemiology, and End Results (SEER) from 1974–2013 revealed that the annual percent change (APC) of total thyroid cancer was 3.6%. During the same period, papillary thyroid cancer (PTC) was the most frequent, and had the highest APC among common histological types. In the Republic of Korea, the incidence of thyroid cancer increased more rapidly, which increased 15-fold from 1993 to 2011^4,5^. The increase in thyroid cancer incidence has also been reported in other countries like Italy, France, England, and Australia.

According to the SEER data, the APC of thyroid microcarcinoma was as high as 9.3%^6^. The number of PTMCs increased in all age groups, and PTMC has become the most common thyroid tumor in patients older than 45 years in the USA^7^. In northwestern Spain, the incidence of PTMC increased from 16.7% in 1978 to 43% in 2001^8^. The proportion of PTMC and non-microcarcinoma both increased. Similar increases of PTMC incidences were also seen worldwide^5,9,10^. However, the worldwide rise in the incidences of thyroid cancer has not been followed by an increase in disease-specific mortality^11^. The mortality of thyroid cancer has remained stable over time.

## AS in thyroid cancer

### Definition of AS

AS refers to the life-long application of meticulous diagnostic modalities to check for changes in the status of a disease without immediate therapeutic measures until the progression of the disease is evident. AS is a treatment option that involves regular testing and assessment of signs of cancer progression, followed by active treatment if the cancer progresses^12^. Using AS means that the cancer is evaluated over time to determine if it starts to progress to a certain point, at which time treatment is necessary. AS has been applied to very low risk cancers such as prostate cancer^12^, and this type of cancer is the most widely used in AS research.

### Initiation of AS in low risk PTMCs

In 1993, Dr. Miyauchi first hypothesized that most PTMCs would remain small and would not develop into clinically significant disease or progression^13^. He proposed that AS was the best strategy to identify low risk PTMCs, which remain latent without disease progression. PTMCs without clinical evidence of metastases or local invasion and without convincing cytological or molecular aggressive characteristics were considered as low risk tumors^2^. Dr. Miyauchi hypothesized that observation without immediate surgery could determine progressive PTMC, and that if slight progression was identified, a rescue surgery should be the effective treatment. The author believed that AS would result in more good than harm for PTMC patients. Cancer Institute Hospital of JFCR (Tokyo, Japan) started a similar AS trial in 1995^14^. To date, most of the findings with AS of PTMC were reported by these two institutes.

### Candidate selection criteria for AS

In 2015, ATA guidelines recommended AS as a reasonable choice for PTMC treatment, and established its criteria for PTC risk classification^2^. In the Republic of Korea, The Korean Thyroid Association (KTA) recommended the same guidelines as the ATA guidelines^15^. The Chinese Association of Thyroid Oncology (CATO) also proposed a selection criteria for AS, which first included information about the family history of thyroid carcinoma and a history of neck exposure to radiation during childhood or adolescence^16^. These guidelines stated that AS “can be considered” as an alternative to immediate surgery in patients with very low risk tumors. However, these guidelines were established for the management of thyroid carcinoma, but not for AS of PTMCs, until Brito et al.^17^ from the Memoria Sloan Kettering Cancer Center published a clinical framework, which established the risk stratification of AS for PTMCs. The stratification divided PTMCs into ideal, appropriate, and inappropriate cancers for AS, by comprehensively evaluating tumor ultrasound characteristics, patient characteristics, and medical team characteristics. Recently, Tuttle et al.^18^ updated the basic framework previously proposed and made some minor modifications based on their ongoing experience and other published data. They stated that PTCs with tumor sizes between 1.0 and 1.5 cm were also acceptable for appropriate AS participates. Moreover, isolated *BRAF* V600E mutations were considered appropriate for AS. For the inappropriate AS criteria, they considered patients with high risk molecular profiles (e.g., multiple mutations or driver mutations)^19^ and patients whose tumor sizes increased (3 mm in diameter or 50% in volume) during a very short time^20^ to be at potentially higher risk of disease progression, and recommended excluding these patients from AS.

The Kuma Hospital recently published a contraindication for the AS of PTMC, which divided the contraindications into two categories. One was the presence of clinical node metastasis, distant metastasis at diagnosis, vocal cord paralysis due to invasion of the recurrent laryngeal nerve, or high grade malignancy or cytology. The other included PTMCs attached to the trachea or located along the path of the recurrent laryngeal nerve^21^, which was based on a previous study investigating the relationship between the possibility of tracheal invasion and the angles formed by the tumor and tracheal surfaces^22^. At the Kuma Hospital, PTMCs located within the thyroid lobe were ideal candidates for AS; moreover, those with minimal extra thyroid extension at the anterior or lateral surface of the thyroid were not considered as contraindications for AS^21^.

However, other guidelines like that of the American Association of Clinical Endocrinologists and British Thyroid Association do not suggest AS in the management of PTMC, so AS as an alternative to surgery remains controversial^23,24^ (detailed criteria of different guidelines for AS are shown in the supplementary materials).

Once a patient is enrolled in the AS program, informed consent should be signed after an explanation of all the pros and cons of AS versus immediate surgery. We compared the different guidelines and different studies of adult AS candidate selection criteria as shown in **Table 1**.

### Follow-up methods and schedules

For patients who give consent to enroll in AS, their PTMCs should be closely monitored according to the following protocols. Even though the follow-up protocols may have slight differences, a 6-month follow-up examination is suggested by most protocols. Particularly at Kuma Hospital, when a patient is identified as an AS candidate, he/she was assigned to a 6-month follow-up of ultrasound analysis after AS initiation. If no progressions were detected during the first follow-up, the next visit was scheduled 1 year later and then every 1 year thereafter. In the USA, the AS approach required ultrasound analysis of the neck every 6 months. After the first 2-year observation, if disease stability was documented, ultrasound exams were conducted every 1–2 year(s). Thyroid function tests were also suggested every year.

### Exploring AS in low risk PTMCs: worldwide outcomes

AS was introduced as an optimal management for all adult patients with low risk PTMCs for several years. At present, most studies involving AS for PTMC were conducted in Japan, and most conclusions were obtained from Japanese data. So far, none of the patients who underwent AS were reported to have life-threatening distant metastases or died from PTMC. The small minority of patients whose PTMC progressed during AS were treated with an appropriate surgery. The outcomes of AS and immediate surgery results were also excellent. Other countries also determined the usefulness of AS in PTMC treatment. Some studies obtained the same results as Kuma Hospital, and suggested AS as a first-line treatment for PTMC, while other studies did not make this recommendation. Based on this limited data, AS was not equally accepted by all clinicians in the world. Memorial Sloan Kettering Cancer Center suggested that accurate risk stratification should be taken for patients who underwent AS and initial risk stratification, and ongoing dynamic risk assessments should individualize treatment for each thyroid cancer patient. Reports from Australia also stated the concerns of clinicians regarding AS. We summarize the main finding of AS worldwide in **Table 2**.

### Japan

Ito et al.^25^ from Kuma Hospital first reported an observation trial of PTMC. They enrolled 162 PTMC patients undergoing AS with a median follow-up time of 46.5 months. During the follow-up period, 70% of the tumors showed a stable disease, while 27.5% showed enlargement in size and 1.2% had lateral neck lymph node metastasis. Subsequently, the investigators renewed their research in 2010 and 2014. In studies published in 2010, 340 patients undergoing observation were followed-up for an average of 74 months, which showed an enlargement of ≥ 3 mm in 6.4% (at 5 years) and 15.9% (at 10 years), and novel nodal metastasis was detected in 1.4% (at 5 years) and 3.4% (at 10 years)^26^. Their report in 2014 found that PTMC in young patients may be more progressive than in older patients. Older patients with PTMC may therefore be the best candidates for observation^27^.

Studies from the Cancer Institute Hospital of JFCR in Japan also showed similar results. In this study, 230 patients were enrolled, and 90% of PTMCs either did not change or decreased in size, when compared to their initial sizes at diagnosis; while 7% showed size enlargement, and 1% showed novel node metastasis during AS^14^. Furthermore, Sakai et al.^28^ conducted a prospective trial of AS for 61 patients with T1bN0M0 PTC and 360 patients with T1aN0M0 PTC. After AS, 8% of the patients with T1aN0M0 PTC and 7% of the patients with T1bN0M0 showed an increase in tumor size. The development of lymph node metastasis was seen in 1% of the patients with T1aN0M0 and 3% of the patients with T1bN0M0. Insignificant differences were found between T1a and T1b. They suggested that AS was an option for selected patients with T1bN0M0 PTC. Most recently, Miyauchi et al.^29^ reported tumor volume changes over time during AS of PTMCs, which showed a decrease in tumor volume in 17% of the tumors.

### USA

Investigators in the USA have initiated AS as a management strategy for low risk PTMC patients since 2014. There are still a small number of AS cohort studies currently in progress. The first report of AS in the USA was from the Memorial Sloan Kettering Cancer Center, which observed 291 patients for a median AS of 25 months. They found no regional or distant metastasis during the AS, and only 3.8% (11 of 291) showed tumor growth in tumor diameter (> 3 mm). They suggested that a 3 mm increase in maximal dimension or a 50% increase in tumor volume may be allowed to continue if the nodule was small, confined to the thyroid, and grew very slowly over time (i.e., tumor volume doubling times greater than 3–5 years)^20^. The progression of a tumor might be more suitable to evaluation by size enlargement if included with a time variable (tumor volume doubling time).

### Republic of Korea

A study from the Asan Medical Center enrolled 2,863 PTMC patients, who were assigned into three groups according to the surgery delay periods (≤ 6 months, 6–12 months, and = 12 months). They found that delayed surgery was not associated with a higher risk of recurrence, when compared to immediate surgery^30^. Later, a multi-center retrospective cohort study screening 370 PTMCs from the Asan Medical Center, Samsung Medical Center, and The Catholic University of Korea Seoul ST. Mary’s Hospital was followed-up for more than 1 year. They found a significant number of increased PTMCs during AS (6.9% at 2 years, 17.3% at 3 years, 28.3% at 4 years, 36.2% at 5 years, and 47% at 6 years), and tumor volume changes were a better method of evaluation than tumor diameters^31^. A 50% increase in tumor volume was too sensitive to determine the time for conversion to surgery. However, the use of volume or diameter to evaluate timing for surgery still needs more study.

### Australia

Two studies from Australia discussed physicians’ and patients’ concerns regarding AS. Nickel et al.^32^ using semi-structured qualitative questionnaires to interview 25 PTMC patients, which stated that clinicians may not be ready to accept AS until the appearance of much stronger evidence. Both these studies suggested that PTMCs were being over diagnosed, and management guidelines are now recommending more conservative management options for these lesions when making treatment decisions^33^. Dr. Miyauchi from Kuma Hospital explained that a PTMC patient who selected immediate surgery might be made vulnerable by a second surgery if lymph node metastasis was found after the first surgery^13^. The outcomes of 1 or 2 surgeries were both excellent. However, patients and clinicians remained concerned about the delay in surgery by AS, which may result in disease progression and the appearance of lymph node metastasis. Based on this limited data, AS has not been equally accepted by all physicians in the world.

### China

Qian et al.^34^ evaluated PTMC patients using two screening criteria for AS (the CATO and Kuma criteria). A total of 72.6% of the 778 enrolled patients met the Kuma criteria, while only 14.4% met the CATO criteria. In this study, the CATO low risk subgroup had lower recurrence and longer disease-free survival than the CATO high risk subgroup. However, no difference was found between the Kuma low risk and high risk groups. They suggested that the CATO criteria, which included a family history of thyroid carcinoma and a history of neck exposure to radiation during childhood or adolescence, was more strict and could be more suitable for Chinese PTMC patients who selected AS.

### Other countries

Sanabria et al.^35^ examined 57 Colombian PTMC patients (Bethesda V to VI) who were treated using AS and found that the tumors of 2 (3.5%) patients grew more than 3 mm. In this study, PTMCs with tumor sizes between 1–1.5 cm were enrolled in AS. This was the first study that provided AS data from Latin America.

Molinaro et al.^36^ used a prospective-observational study to evaluate the feasibility of AS in PTMC patients in Italy. After a median follow-up of 19 months, only 3% of PTMC patients showed disease progression. They concluded that AS was an achievable and effective alternative management strategy for PTMC patients in Italy.

## Some concerns about AS

There are several conflicting situations regarding AS of PTMCs, which include the usage of a biopsy, definition of “disease progression,” thyrotropin-stimulating hormone (TSH) suppression, medical cost analysis, and quality of life (QoL) evaluation. We summarized the controversies of these topics and suggested that a large-scale, well-designed cohort of low risk PTMC patients under AS is still needed to resolve these issues.

### Necessity of cytological or pathological confirmation

Whether tumors with a high suspicion of PTMC undergo cytological or pathological confirmation depends on the country. In Japan, guidelines established by JAES/JSTS suggested that PTMC should be diagnosed by cytological or pathological confirmations. Kuma Hospital insisted on a biopsy for two reasons. First, if the cytological test was not performed at Kuma Hospital, the patient might visit other physicians who might perform cytological tests, and they might suspect that Kuma Hospital had missed the diagnosis of cancer, and suggest immediate thyroid surgery. The other reason was that without a cytological diagnosis, patients may lose the opportunity for AS outside of Kuma Hospital. In the Memorial Sloan Kettering Cancer Center in the USA, cytological or pathological confirmation was not a requirement for AS of PTMC. When the patients were suspected of malignant disease after clinical or ultrasound imaging tests, AS was also provided. In their AS management program, a biopsy was not required for the enrollment of patients^17^. However, in a later report from the Memorial Sloan Kettering Cancer Center, which observed 291 patients for an AS of 25 months (median), the patients enrolled in the study had PTC (Bethesda category IV) or suspicious PTC (Bethesda V) with suspicious ultrasound imaging characteristics^20^. The 2015 ATA guidelines do not recommend cytological examination for PTMC for tumors < 10 mm (even if they have suspicious ultrasound features), unless they are associated with clinical symptoms or lymphadenopathy^2^. Until now, most of the long-term follow-up data of AS were based on PTMCs that were cytologically diagnosed. Researchers also suggested that it was reasonable to apply these selection criteria to sonographically suspicious cases, without a biopsy^37^. Without a cytological confirmation of PTMC, patients with suspicious nodules would be undergoing an ultrasound image analysis at follow-up. However, ultrasound imaging has its limitations in distinguishing small medullary thyroid carcinomas from PTMCs^38^; therefore a calcitonin measurement might be helpful to distinguish small medullary thyroid carcinomas from PTMCs. For patients with suspicious nodules = 5 mm, a cytological or pathological confirmation test may be suggested before enrollment for AS. Cytological diagnosis by an experienced clinician is also important. For those patients with suspicious nodules <5 mm, cytological or pathological confirmation is not recommended, so an ultrasound follow-up may be the best choice.

### Progression evaluation

#### Diameter or volume

The first report of the use of volume was from the Memorial Sloan Kettering Cancer Center, which showed only 3.8% (11 of 291) of the patients had growth with a tumor diameter = 3 mm. They discovered that tumor volume was a more sensitive marker for tumor enlargement than tumor diameter^20^. However, to terminate AS and start therapeutic intervention, the use of volume or diameter is still controversial. Kwon et al.^39^ at the Asan Medical Center reported the results from a retrospective cohort study in 2017. They found that using the change in tumor volume was more sensitive in detecting the growth of tumors than using the change in maximum diameter. They enrolled 192 PTMC patients, who were cytopathologically diagnosed. After more than 1 year of AS, the cohort had a median follow-up of 30 months. A total of 27 PTMC patients showed an increase in tumor size, and 1 patient had newly apparent lymph node metastasis, while 33 PTMC patients had decreasing tumor size. More recently, a multi-center cohort from the Republic of Korea also suggested that tumor volume change may be more sensitive to evaluate tumor growth than use of the tumor diameter^31^. After 32.5 months of follow-up, 23.2% (*n* = 86) of the patients were shown to have an increase in volume, and 3.5% (*n* = 13) of the patients were shown to have an increase in the maximal diameter. Tumor volume was calculated by multiplying three diameters, so the increase in volume was easier to detect than an increase in diameter. However, the investigators found a 50% increase in tumor volume was too sensitive to determine the time for conversion surgery, so the use of volume or diameter to evaluate the timing for conversion surgery still needs more study. Further studies are still needed for the evaluation of tumor growth during AS. Moreover, no matter whether diameter or volume is used in evaluating the progression of a tumor, we also need to consider the time it takes to distinguish tumors that increase in size over decades from those that increase in size over a short time^20^.

Ito et al.^40^ from Kuma Hospital recently reviewed 824 PTMC patients undergoing AS between 2005 and 2011. This study presented important findings that growth activity decreased in most PTMCs after enlargement, and that the tumors shrank in certain cases. Accordingly, the necessity of immediate surgery after reaching the point of enlargement should be considered. At present, some studies set the upper limit of tumor size at 13 mm for AS. Further investigations are needed to determine whether this threshold is appropriate.

#### Pregnancy

The 2017 ATA guidelines on the management of thyroid nodules and thyroid cancer during pregnancy suggested that ultrasound monitoring of the thyroid should be performed each trimester during pregnancy in pregnant PTMC patients who undergo AS^41^. Shindo et al.^42^ reported on 9 women with PTMC who became pregnant during AS and compared their outcomes to 27 age-matched non-pregnant women. Growth of the tumor occurred in 44.4% (4 of 9 patients) of pregnant patients, whereas it occurred only in 11.1% (3 of 27 patients) of the control patients (*P* = 0.0497). However, this study was found to have a large selection bias. After reevaluation of the data in the Entire Patient Series at Kuma Hospital from 1993 to 2013, investigators found that 8% (4 of 51) of the patients showed enlargement of PTMCs by ≥ 3 mm, 90% showed stable disease, and none showed a novel appearance of lymph node metastasis^43^. A multi-center cohort study from the Republic of Korea enrolled 370 patients, which included 5 pregnant patients with PTMCs. After a follow-up of 18.3 months, 2 of 5 patients had significant increases in size. Besides these 2 patients who showed progression of the disease during pregnancy, another 2 patients also chose to undergo surgery because of anxiety^31^. Presently, only these studies involved AS during pregnancy. Whether AS is safe during pregnancy is still under discussion, so a large well-designed study is needed for further evaluation.

#### Age

The estimated lifetime disease progression probabilities of PTMC during AS vary greatly according to age. The lifetime probability for PTMC progression may be 5%–10% in patients diagnosed after an age of 60 years, 15%–30% in patients diagnosed in their 40’s and 50’s, and as high as 40%–60% in patients diagnosed in their 20’s and 30’s^44^. Based on these findings, the Memorial Sloan Kettering Cancer Center suggested that older patients (≥ 60 years of age) were ideal candidates for AS, middle aged patients (18–59 years of age) were appropriate candidate, and young patients (<18 years of age) were inappropriate candidates^17^. However, the guidelines did not mention the appropriate age cut-off for AS.

#### Progression markers

PTMC is the most common PTC, with the majority of PTMCs following an indolent course, whereas the other cancers show disease progression. Unfortunately, there is no progression marker to distinguish the indolent PTMCs and aggressive PTMCs. AS is the only way to distinguish these two groups of patients. Hirokawa et al.^45^ investigated the possibility that pathological characteristics were progression markers of PTMCs during the surveillance period. The Ki-67 labeling index was found to be higher in enlarged PTMCs compared to those in non-enlarged PTMCs during AS. Kim et al.^46^ analyzed 127 PTMC patients who underwent AS and found that high serum TSH levels were associated with progression of PTMC during AS. Although routine molecular analysis was not required for AS, the wide use of molecular profiling of thyroid nodules identified some patients with gene mutations, which provided evidence for the use of risk assessment markers. As reported, the *BRAF* V600E mutation alone was a sensitive but not specific marker of PTC recurrence and mortality^2^. Based on these results, the Memorial Sloan Kettering Cancer Center suggested that isolated *BRAF* V600E mutations are considered appropriate for AS^18^. When a patient is diagnosed with co-occurring mutations (e.g., *BRAF* and *TERT*, *RAS* and *TERT*, and *BRAF* or *RAS* with *TP53*, *PIK3CA*, or *AKT1* mutations) they usually have an unfavorable outcome^19^. The Memorial Sloan Kettering Cancer Center suggested that patients carrying high risk co-occurrence mutations are inappropriate for AS^18^. However, the specific role of molecular profiling in identifying tumor aggressiveness in PTMC remains to be elucidated, although a study of these molecular profiling markers would help in the selection of appropriate low risk PTMC patients for AS.

The age decade-specific disease progression rates at 10 years of AS decreased from 36.9% in the 20’s, to 3.5% in the 70’s^44^. The estimated lifetime disease progression probabilities of PTMC during AS varied greatly according to age. AS was thought to be the only method to recognize progressive PTMCs. However, based on large-scale studies, investigators have found that the only progression marker is patient age.

### Medical costs and cost-effectiveness analysis

The medical costs of immediate surgery ($8,437 US dollars) was 4.1 times higher than the costs of AS for a 10 year management ($2,052 US dollars), including conversion surgery and the salvage surgery cost^47^. Lang et al.^48^ first examined the cost-effectiveness of the AS strategy for PTMC. They found that AS was more cost-effective during the first 16 years than immediate surgery. When only considering costs, after 17 years, AS costs more than immediate surgery, although the cost of immediate surgery is significantly higher than AS at the very beginning. However, AS cost more over time because of continuing examinations and the accumulating possibility of a more expensive “delay” surgery. When considering quality-adjusted life years, AS is more effective than immediate surgery because the AS strategy may result in less complications than immediate surgery. Investigators used cost-effectiveness analyses using Markov models for AS and hemithyroidectomy. They found that for patients under AS, the cost-effectiveness of hemithyroidectomy decreased both the QoL and life expectancy^49^. Lin et al.^50^ compared the costs of surgery versus hypothetic AS for PTMC in an Australian cohort of 349 patients. They found that the estimated cost of PTMC surgical treatment ($10,226 Australian dollars) was equivalent to the cost of 16.2 years of AS ($756 Australian dollars/year). In Australia, surgery might have a long-term economic advantage for younger PTMC patients. The cost of surgery varied greatly in different countries, so the conclusions obtained from cost-benefit analyses in different countries can only be applied to the clinical practice in those countries.

### TSH suppression

TSH suppression is a common strategy to prevent papillary carcinoma recurrence or progression. However, there has been no large-scale report, which examined the efficacy of TSH suppression of PTMC. Some physicians at Kuma Hospital prefer to perform mild thyrotropin suppression, which means setting serum TSH levels lower than the lower normal limit. Based on the judgement of the physician, only 51 patients out of 1,235 patients underwent TSH suppression, and most of the PTMC patients enrolled in this TSH suppression study were clinically stable^27^. Nevertheless, Sugitani et al.^14^ reported that serum TSH level was not associated with progression during their observation of PTMC. Because TSH suppression may induce osteoporosis in elderly female patients, 2015 ATA guidelines suggested that TSH suppression was not routinely recommended in low risk differentiated thyroid cancer after surgery, especially for older patients. TSH suppression may therefore be more suitable for PTMCs in young patients, whose disease is slightly more progressive than the more stable PTMC in elderly patients. However, further studies with more samples are needed to obtain more definitive conclusions.

### QoL

QoL is a very important issue for AS of PTMC. In prostate cancer studies, where AS is frequently used, the QoL was similar between the AS group and the immediate surgery group^51^. Investigators reported that anxiety and fear may decrease during AS, and a greater complication rate may contribute to a lower QoL. Oda et al.^52^ investigated the unfavorable events between AS and immediate surgery of PTMC patients, by studying 179 PTMC patients who underwent AS and 94 PTMC patients who underwent immediate surgery. The results showed that surgical complications (including temporary vocal cord paralysis, temporary/permanent hypoparathyroidism, skin surgical scar, and/or postsurgical hematoma) were more frequent in the immediate surgery group. However, these studies were found to contain misleading results because of the use of an inappropriate study population. Oda et al. compared the complication rate between immediate surgery and the total AS group. When excluding patients without surgery and considering only those who received delayed surgery, they reanalyzed the data and showed that the complication rate was higher in the patients who underwent delayed surgery when compared with those who underwent immediate surgery. The reason for this result was that patients may have had a chance of developing lymph node metastasis during AS, resulting in more extensive surgery, which may have a greater chance of surgical complications. In summary, patients do not suffer from surgical complications if they select AS and delayed surgery; however, they might experience higher rates of surgical complications if they are treated with delayed surgery.

Kong et al.^53^ recently evaluated the QoL of 203 patients who selected AS and 192 patients who underwent immediate surgery, using interim analyses of a multi-center prospective cohort study in the Republic of Korea (MAeSTro). The QoL of the two groups were evaluated by a thyroid-specific QoL questionnaire, and the evaluations were conducted both at the time of diagnosis and after a median of 8 months of follow-up. They found that the AS group had better psychological health at baseline, and the physical and psychological health of the AS group during the follow-up were better than that of the immediate surgery group. Davies et al.^54^ described the patients’ burden of cancer concern in the longest-standing and largest PTMC AS cohort at Kuma Hospital (Japan). By surveying 243 patients with AS, it was found that cancer concerns were common among patients with AS. The number of patients who stated that they did not worry increased from 14% at the time of diagnosis to 25% after 3 years of follow-up. Cancer concerns of AS PTMC patients and surgery PTMC patients were similar. Cancer concerns should not necessarily be viewed as uniformly prohibitive to successful AS in thyroid cancer. However, the follow-up time was relatively short for the current study, so studies with longer follow-up periods are warranted.

## Discussion

Because of concerns of overdiagnosis and overtreatment, AS has been introduced as a strategy for low risk PTMC patients. The worldwide results of AS protocols for low risk PTMC patients have mostly been reported from two Japanese institutions, Kuma Hospital in Kobe and the Cancer Institute Hospital of JFCR in Tokyo^14,25–27,43^. Although these findings have assured some clinicians that AS was safe, and that AS was more suitable than immediate surgery as the first-line management for low risk PTMC patients, the biological characteristics of PTMC patients in countries other than Japan might differ. Well-designed large-scale multi-center cohort studies are still needed to obtain more definitive conclusions.

In the absence of accurate methods to distinguish stable PTMC from aggressive PTMC, observation strategies such as AS have been used by most clinicians. Identifying biomarkers would help in the decision-making process. Investigators have identified some markers of progression, but none have been established. The omics approaches have gained much attention, which include genomics, transcriptomics, proteomics, and metabolomics^55,56^. The omics studies found several biomarkers for cancer diagnosis and prognosis by analyzing DNA sequences, gene expression, protein expression, metabolites, and related biochemical reactions^57^. In addition, integration of genomics, transcriptomics, proteomics, and metabolomics of PTMC will provide better insight into an understanding of the biochemical cause of PTMC.

At present, a good prognosis of thyroid cancer mainly refers to the low mortality rate, and this is because of combined treatment [surgery, radioactive iodine (RAI), and TSH suppression therapy]. However, the prognosis of PTMC is not always accurate in terms of lymph node metastasis, recurrence, or extrathyroidal invasion. During the PTMC observation period, there is still no consensus on how to determine the follow-up interval and which follow-up indicators should be included. Different guidelines may have different criteria of PTMC enrollment and different criteria of patient exclusion for AS. Whether ultrasound imaging or other neck imaging modalities such as computed tomography^58^ can detect lymph node metastasis accurately, and how to correct the variances between different ultrasound operators when analyzing the size of tumors are still not completely established.

Moreover, clinicians only deal directly with patients’ physiological parameters. Psychological changes and the mental state of patients when facing PTMC are also important determinants of the application of observation strategies. Recently, Davies et al.^54^ reported that cancer concerns were found among patients with AS, which decreased over time, and patients expressed satisfaction with their decision in choosing AS. In addition, a lack of necessary psychological support and counseling for patients during PTMC observation will also affect the QoL of patients, thereby affecting the sustainability and effectiveness of the strategy.

In summary, considering the clinical data obtained, clinicians should discuss alternative treatment options with low risk PTMC patients and respect their choices. Clinicians may introduce the advantages and disadvantages of AS to low risk PTMC patients, and carefully address the concerns of the patients. For PTMC patients who are willing to accept AS, clinicians should implement a standardized clinical practice mode, use informed consent forms, establish a follow-up plan according to ethical requirements, establish a specialized follow-up team, accumulate follow-up observation experience, and provide psychological counseling and support to patients.

## Conclusions

The efficacy of AS for low risk PTMC patients mostly depends on the accurate evaluation of patient risk. Clinicians may adopt measures like dynamic monitoring, risk stratification, and personal follow-up schedules to minimize these potential risks. However, a large-scale, well-designed cohort of low risk PTMC patients who undertake AS is still needed to ensure its long-term safety, as well as to identify prognostic and diagnostic markers for tumor progression.

## Figures and Tables

**Table 1:** Comparison of different guidelines and different studies for the active surveillance (AS) candidates selection criteria

Guideline	Biopsy	Tumor characteristics								Patient characteristics				Medical team characteristics		
		Number of nodules	Size of nodule	Margin	Location	Stability	Extrathyroidal extension	cN0	cM0	Age^¶^	Willing	Life-threatening comorbidities	Risk factors	Multidisciplinary team	US	Follow-up
ATA	Yes						Yes	Yes	Yes			Yes			Yes	Yes
KTA	Yes						Yes	Yes	Yes			Yes			Yes	Yes
CATO	Yes		Yes (≤5 mm)				Yes	Yes	Yes		Yes	Yes	Yes (family history; radiation)		Yes	Yes
Memorial Sloan Kettering Cancer Center, USA	Not necessary^†^	Yes (multifocal)	Yes (<1 cm ideal; 1–1.5 cm appropriate)	Yes	Yes^‡^	Yes	Yes	Yes	Yes	Yes	Yes	Yes	Yes (molecular profile)	Yes	Yes	Yes
Kuma Hospital, Japan	Yes	Yes (multifocal)			Yes^§^		Yes	Yes	Yes		Yes	Yes			Yes	Yes
Cancer Institute Hospital of JFCR, Japan	Yes	Yes (multifocal)	Yes (<1 cm; 1–2 cm T1bN0M0)				Yes	Yes	Yes		Yes	Yes			Yes	Yes
Asan Medical Center, Korea	Yes						Yes	Yes	Yes			Yes			Yes	Yes
University Hospital of Pisa, Italy	Yes	Yes (single)	Yes (≤1.3 cm)				Yes	Yes	Yes	Yes			Yes (thyroid dysfunction)		Yes	Yes

^†^In the later publication of the Memorial Sloan Kettering Cancer Center, which observed 291 patients for an active surveillance (AS) of 25 months (median), the patients enrolled in the study had papillary thyroid carcinoma (PTC) (Bethesda category IV) or suspicious PTC (Bethesda V) with suspicious ultrasound characteristics. ^‡^Not adjacent to the recurrent laryngeal nerve (RLN). ^§^Including the location of the tumor in relation to the RLN and trachea. ^¶^Although many guidelines did not mention the patient age, they should be established on the premise for management for adult patients.

**Table 2:** Natural history of low risk papillary thyroid microcarcinoma (PTMC): main worldwide findings

Study, year (country)	Institute	Number of patients	Tumor size	Follow-up time (months) mean, median*	Growth rate	LNM rate	Distant metastasis rate
Ito et al., 2014 (Japan)	Kuma Hospital	1235	<1.0 cm	18–227 (60)	4.60%	1.50%	0
Sugitani 2018 (Japan)	Cancer Institute Hospital of JFCR	360	<1.0 cm	6–300 (87.6)	8.00%	1.00%	0
Sugitani 2018 (Japan)	Cancer Institute Hospital of JFCR	61	1.0–2.0 cm	12–204 (94.8)	7.00%	3.00%	0
Tuttle et al., 2017 (United States)	Memorial Sloan Kettering Cancer Center	291	<1.5 cm	6–166 (25*)	3.80%	0	0
Sanabria et al., 2018 (Colombia)	Head and Neck Cancer Center in Medellín	57	<1.5 cm	0–54 (13.3*)	3.50%	0	0
Oh et al., 2018 (Korea)	Asan Medical Center, Samsung Medical Center, The Catholic University of Korea Seoul ST. Mary’s Hospital	370	<1.0 cm	21–47 (32*)	3.50%	1.30%	0
Molinaro et al.,2020 (Italy)	University Hospital of Pisa	93	≤1.3 cm	6–54 (19*)	2.15%	1.07%	0
