# Supplementary material for: Active surveillance as a management strategy for papillary thyroid microcarcinoma
Source: Cancer Biol Med. 2020 Aug 15;17(3):543–54. doi: 10.20892/j.issn.2095-3941.2019.0470 (PMC7476094; doi:10.20892/j.issn.2095-3941.2019.0470)
Supplement: Supplementary file 1 [file cbm-17-543-s001.pdf]

Supplementary materials

Candidate selection criteria for active surveillance (AS) (details of different guidelines)

1. ATA guidelines[2]: patients with very low risk tumors; patients at high surgical risk because of comorbid conditions; patients expected to have a relatively short remaining lifespan; patients with concurrent medical or surgical issues that need to be addressed prior to thyroid surgery.
2. KTA guidelines[15]: the same as the recommended ATA guidelines.
3. CATO guidelines[16]:
   3.1 Patients with non-high risk pathological variants
   3.2 Patients with tumor diameters ≤ 5 mm
   3.3 Patients with tumor distant from the thyroid capsule and not having invaded surrounding tissues
   3.4 Patients without evidence of lymph node or distant metastasis
   3.5 Patients without a family history of thyroid carcinoma
   3.6 Patients without a history of neck exposure to radiation during childhood or adolescence
   3.7 Patients with a low psychological burden who can actively cooperate.
4. Memorial Sloan Kettering Cancer Center[18]:
   Memorial Sloan Kettering Cancer Center developed a framework for the clinical decision making of PTMCs according to three domains (tumor/neck ultrasound characteristics; patient characteristics; medical team characteristics). They divided patients into three kinds of candidate for AS: ideal candidates; appropriate candidates; inappropriate candidates.
   4.1 Ideal candidate
   Tumor/neck ultrasound characteristics: solitary thyroid nodule confined to thyroid; ≤ 1 cm; well-defined tumor margins by ultrasound; surrounded by ≥ 2 mm normal thyroid parenchyma; previous US documenting stability; cN0; cM0.

   Patient characteristics: medical minimalist; older patients (≥ 60 years of age); willing to accept AS understands that future surgery may be necessary (deferred intervention); understands that lymph node metastasis may be identified during the follow-up; compliant with follow-up plans; supportive of significant others (including other members of their health care team); life-threatening comorbidities or medical conditions requiring therapy.

   Medical team characteristics: experienced team; expeditious evaluation by a multidisciplinary team; high quality neck ultrasonography; prospective data collection; tracking/reminder program to ensure proper follow-up.

4.2 Appropriate candidate

Tumor/neck ultrasound characteristics: multifocal papillary microcarcinomas; 1–1.5 cm maximal dimension; subcapsular location not adjacent to the RLN without evidence of extrathyroidal extension; ill-defined tumor margins; background ultrasonographic findings that will make follow-up difficult (thyroiditis, reactive lymph nodes, and multiple other begin appearing thyroid nodules); F-18 fluorodeoxyglucose (FDG) avid PTMC; an isolated *BRAF* V600E mutation.

Patient characteristics: minimalist/maximalist; middle-aged patients (18–59 years of age); extensive family history of papillary thyroid cancer; child bearing potential.

Medical team characteristics: experienced endocrinologist or thyroid surgeon; ultrasonography routinely available.

4.3 Inappropriate candidate:

Tumor/neck ultrasound characteristics: aggressive cytology features (rare); locations adjacent to the RLN/trachea; evidence of extrathyroidal extension; clinical evidence of invasion of the RLN or trachea (rare); N1 disease or M1 disease; high risk molecular profile; demonstrated increase of 3 mm diameter of a 50% increase in tumor volume over a relatively short time period.

Patient characteristics: medical maximalist; young patients (< 18 years of age); unlikely to be compliant with follow-up plans; not willing to accept an observation approach; severe anxiety regrading treatment.

Medical team characteristics: reliable neck ultrasonography not available; little experience with thyroid cancer management.

5. Kuma Hospital (contraindications for AS)[21]:
   High risk features: presence of clinical node metastasis and/or clinical distant metastasis at diagnosis; signs or symptoms of invasion to the recurrent laryngeal nerve or trachea; high grade malignancy on cytology (e.g., tall cell variants and poorly differentiated carcinoma).

   Features rendering AS unsuitable: tumors attaching to the trachea; tumors located in the pathway of the recurrent laryngeal nerve.
